# Deciphering respiratory viral infections by harnessing organ-on-chip technology to explore the gut–lung axis

**DOI:** 10.1098/rsob.240231

**Published:** 2025-03-05

**Authors:** Hristina Koceva, Mona Amiratashani, Parastoo Akbarimoghaddam, Bianca Hoffmann, Gaukhar Zhurgenbayeva, Mark S. Gresnigt, Vanessa Rossetto Marcelino, Christian Eggeling, Marc Thilo Figge, Maria-João Amorim, Alexander S. Mosig

**Affiliations:** ^1^ Institute of Biochemistry II, Jena University Hospital, Jena, Germany; ^2^ Cluster of Excellence Balance of the Microverse, Friedrich-Schiller-University Jena, Jena, Germany; ^3^ Applied Systems Biology, Leibniz Institute for Natural Product Research and Infection Biology, Hans Knöll Institute (HKI), Jena, Germany; ^4^ Leibniz Institute of Photonic Technologies e.V., Member of the Leibniz Centre for Photonics in Infection Research (LPI), Jena, Germany; ^5^ Junior Research Group Adaptive Pathogenicity Strategies, Leibniz Institute for Natural Product Research and Infection Biology, Hans Knöll Institute (HKI), Jena, Germany; ^6^ Melbourne Integrative Genomics, School of BioSciences, University of Melbourne, Parkville, Australia; ^7^ Department of Microbiology and Immunology, The Peter Doherty Institute, University of Melbourne, Parkville, Australia; ^8^ Institute of Applied Optics and Biophysics, Friedrich-Schiller-University Jena, Jena, Germany; ^9^ Jena Center for Soft Matter, Jena, Germany; ^10^ Católica Biomédical Research Centre, Católica Medical School, Universidade Católica Portuguesa, Lisbon, Portugal; ^11^ Center of Sepsis Control and Care, Jena University Hospital, Jena, Germany

**Keywords:** lung, OoC, respiratory, chips, models, viral

## Abstract

The lung microbiome has recently gained attention for potentially affecting respiratory viral infections, including influenza A virus, respiratory syncytial virus (RSV) and SARS-CoV-2. We will discuss the complexities of the lung microenvironment in the context of viral infections and the use of organ-on-chip (OoC) models in replicating the respiratory tract milieu to aid in understanding the role of temporary microbial colonization. Leveraging the innovative capabilities of OoC, particularly through integrating gut and lung models, opens new avenues to understand the mechanisms linking inter-organ crosstalk and respiratory infections. We will discuss technical aspects of OoC lung models, ranging from the selection of cell substrates for extracellular matrix mimicry, mechanical strain, breathing mechanisms and air–liquid interface to the integration of immune cells and use of microscopy tools for algorithm-based image analysis and systems biology to study viral infection *in vitro*. OoC offers exciting new options to study viral infections across host species and to investigate human cellular physiology at a personalized level. This review bridges the gap between complex biological phenomena and the technical prowess of OoC models, providing a comprehensive roadmap for researchers in the field.

**Figure:**
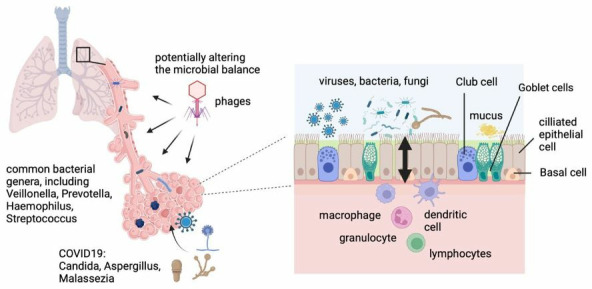

## 1. Influenza, SARS-CoV-2 and RSV: triad of respiratory viruses straining global healthcare systems

Lower respiratory infections remain the leading cause of death among communicable diseases worldwide and, overall, the fourth cause of death [1]. Infections caused by influenza, respiratory syncytial virus (RSV) and SARS-CoV-2 infections contribute the most to yearly mortality and morbidity caused by respiratory viral infections worldwide [2]. Crucially, infections caused by these viruses place significant burdens on healthcare systems, sometimes diminishing the response capacity well beyond the confines of emergency units [3].

Influenza is a contagious respiratory illness associated with a significant clinical burden. Influenza A and B provoke yearly seasonal epidemics, and influenza A virus causes, in addition, occasional pandemics [4]. Pandemic outbreaks are caused by zoonotic events in which influenza A viruses adapted to specific animals cross the host species barrier, establishing in an immunologically naive human population and causing infections worldwide [5,6]. Seasonal epidemic influenza provokes acute respiratory infections in 10% of the world’s population, with 3−5 million cases of severe illness [7] and up to 650 000 fatalities yearly [8,9]. Symptoms linked to influenza virus infection range from a mild respiratory illness affecting the upper respiratory tract, marked by fatigue, fever, cephalalgia, myalgia, sore throat, cough and rhinorrhea, to severe cases or even fatal complications, including pneumonia caused by the influenza virus or secondary bacterial or invasive fungal infections in the lower respiratory tract. Despite most infections occurring in children, and the fact that children play a critical role in disease spread [10], severe cases involve not only young children but also (and mostly) the elderly [11] and immunocompromised individuals with chronic disease and associated comorbidities [12].

RSV is a primary contributor to acute respiratory tract infections in young children, accounting for approximately 30 million cases, 3.6 million hospital admissions and 100 000 deaths annually worldwide [2]. Despite one in approximately 60 healthy infants requiring hospitalization [9], babies up to six months, especially those born prematurely, having chronic lung issues, congenital heart problems and neurological disease, are at higher risk of severe outcomes. Contrastingly with influenza, adults often experience asymptomatic RSV infections but are significant carriers for viral transmission. In adults aged 65 years and older or those with chronic conditions, RSV infection can lead to severe manifestations such as pneumonia or bronchitis [13]. A recent meta-analysis has calculated that annually, approximately 5.2 million cases of acute respiratory infection associated with RSV take place in high-income countries. Within this, there are an estimated 300 000 to 720 000 hospitalizations and 16 000 to 67 000 deaths occurring during hospitalizations among adults aged 60 years and above [14]. In the USA alone, yearly, an estimated 60 000–160 000 older adults are hospitalized, and 6000–10 000 die from RSV infection [15].

In 2019, a new virus, the severe acute respiratory syndrome coronavirus 2 (SARS-CoV-2), appeared in Asia, causing severe pneumonia in humans [16,17]. In March 2020, the World Health Organization declared coronavirus disease 19 (COVID-19) caused by the SARS-CoV-2 a pandemic of global concern. In 3 years, SARS-CoV-2 has caused approximately 677 million infections and 7 million deaths worldwide [18]. Importantly, the disproportional number of people requiring hospital care has put global health systems under immense stress. Despite efforts to keep services active, health structures were unable to provide appropriate care to patients to both respond to the crises and maintain other essential medical care [19]. The challenges in handling the pandemic have prompted reconsidering strategies to enhance healthcare resilience and readiness for unforeseen crises. Clinical manifestations of COVID-19 range from being asymptomatic to critical illness, including death, with most people clearing the infection after 8−14 days. Although SARS-CoV-2 has been shown to infect people of all ages indiscriminately, disease outcomes are heavily dependent on age and associated comorbidities, as recognized soon after the virus emerged in the human population [20]. People at high risk of developing severe disease include the elderly, the immunocompromised, and people with chronic diseases such as hypertension, type II diabetes mellitus, chronic kidney disease, obesity and cardiovascular disease [21].

Despite availability of effective antivirals and preventive measures for all viruses, including vaccines, the burden of any of these viruses is underestimated due to secondary bacterial and fungal infections [22,23], and extrapulmonary complications, including cardiovascular, neurological, renal, ocular and/or hepatic issues [24–28]. Given the high mortality and morbidity associated with these viruses, the increase in antibiotic resistance of bacteria and fungi, and the pandemic potential of SARS-CoV-2 and influenza A virus [29], a better understanding of how these viruses cause pulmonary and extrapulmonary disease is pivotal for developing improved treatment strategies. Recently, it became apparent that inter-organ crosstalk plays a key role in maintaining host homeostasis and regulating susceptibility to respiratory disease [30–32]. The mechanisms behind inter-organ signalling in viral infections are far from understood and must be further explored to get a picture of how viruses affect the entire organism. Here, we discuss the promising advances in organ-on-chip (OoC) technology to study human tissue response to viral challenges *in vitro*.

## 2. The lung microbiome: shaping host responses to viral infections

Due to limitations in microbial culture techniques, the healthy lung was long considered a sterile organ. Culture-independent approaches, such as 16S RNA sequencing and shotgun metagenomics, reveal that the lung hosts a distinct microbial community, including bacteria, archaea, fungi, protozoa and viruses [33]. These techniques help analyse microbial community features based on host factors like comorbidities or infections [34,35]. While culture-independent methods also capture DNA from dead microorganisms, culture-based methods can provide information on bacterial viability, though many species remain unculturable. Hence, it is valuable to utilize both culture-based and culture-independent techniques to study the lung microbiome accurately [36,37].

The microbiome’s composition is ecologically dynamic and could be easily altered by different factors. These factors include microbial immigration (via microaspiration from the upper respiratory tract), microbial emigration or clearance, and local microbe replication. These factors could lead to a state of dysbiosis, a condition where the balance and composition of the beneficial intestinal microbes are disrupted, which could lead to a compromised gut barrier function. Several studies have shown a link between dysbiosis in the lung microbiome and disease or increased disease susceptibility [37,38].

The lung microbiome has been extensively studied, revealing that changes in its composition are linked to various clinical indicators such as disease severity, exacerbation, phenotype, endotype, inflammation and mortality [39]. For instance, bacterial diversity increases in the upper respiratory tract during influenza A and B infections, altering the abundance of genera like *Staphylococcus*, *Bacteroides*, *Haemophilus* and *Fusobacteria* [40]. These microbial shifts can enhance the risk of bacterial pneumonia during viral infections [41]. Studies have shown that during influenza infection, there is a high abundance of *Streptococcus pneumoniae* and *Staphylococcus aureus* in the nose and throat [42]. Similar increases in pneumococcal density are observed during rhinovirus and RSV infections [41]. Table 1 shows an example of culturable microorganisms found in the lung microbiome, which can be subject to change in composition in healthy and diseased conditions.

**Table 1.** Examples of microorganisms found in the lung microbiome.

microorganism	genus	culture	reference
bacteria	*Pseudomonas*	*Pseudomonas* species are aerobic, Gram-negative bacteria that can be cultured on various standard laboratory media	[43–46]
	*Streptococcus*	Gram-positive bacteria that grow well on blood agar, where they typically produce characteristic haemolysis patterns (alpha, beta or gamma haemolysis)	
	*Proteus*	Gram-negative bacteria that can be cultured on standard laboratory media	
	*Clostridium*	Gram-positive bacteria that require specific anaerobic conditions for culturing; can be cultured using anaerobic jars or chambers with media such as thioglycolate broth or cooked meat medium	
	*Haemophilus*	*Haemophilus* species are Gram-negative bacteria that require enriched media containing growth factors	
	*Veillonella*	Gram-negative cocci that can be cultured in anaerobic conditions using media like blood agar	
	*Porphyromonas*	Gram-negative bacteria that require enriched, anaerobic culture conditions, often using blood agar supplemented with hemin and vitamin K	
	*Ralstonia*	Gram-negative bacteria that can be cultured on standard laboratory media like nutrient agar	[47]
	*Bosea*	Gram-negative bacteria that can grow on various standard media under aerobic conditions	[47,48]
	Enterobacteriaceae	Gram-negative bacteria (e.g. *Escherichia*, *Klebsiella*) that are easily cultured on a variety of media, such as MacConkey agar, nutrient agar and blood agar	[47,49]
	*Methylobacterium*	Gram-negative, aerobic bacteria that can grow on standard media like nutrient agar, often with low concentrations of methanol as a carbon source	[47,50]
fungi	*Candida*	fungi that grow well on Sabouraud dextrose agar and can be cultured from clinical specimens like blood and mucosal surfaces	[51,52]
	*Saccharomyces*	yeasts that grow well on a variety of laboratory media, such as yeast extract peptone dextrose (YPD) agar	[53]
	*Penicillium*	filamentous fungi that can be cultured on media like Sabouraud dextrose agar or potato dextrose agar	[52,54]
	*Aspergillus*	filamentous fungi that grow well on Sabouraud dextrose agar or potato dextrose agar	[55,56]
	Davidiellaceae	can be cultured on fungal growth media	[57,58]
	*Eurotium*	fungi that can be cultured on media such as Sabouraud dextrose agar or malt extract agar	[52,54]
viruses	Paramyxoviridae	family includes viruses, such as the respiratory syncytial virus (RSV), which require living cells (e.g. mammalian cell cultures) for propagation	[59]
	Picornaviridae	family includes viruses like rhinoviruses, which must be grown in specific cell cultures	[60,61]
	Orthomyxoviridae	family includes influenza viruses, which require cell culture systems (e.g. chicken embryos or mammalian cell lines) for growth	[62]
	Alpha papillomavirus	require living cells and specific conditions to replicate	[63]

These conclusions were primarily drawn from sequencing human samples and correlating the findings with clinical data. This approach is necessary because, while mouse models are invaluable for understanding immunology, they differ significantly from humans in various aspects of the immune system, which complicates direct extrapolation [64,65]. Differences include the presence of certain immune cells and receptors, expression levels of cytokines and chemokines, and variations in microbiota composition [64,66]. For example, only about 10.8% of gut microbiota species overlap between humans and mice, with some studies estimating as low as 2.58% [66–68]. Microbial metabolite production, such as short-chain fatty acids (SCFAs), also differs between species, impacting immune response modulation in humanized mouse models [69]. These distinctions challenge using mice as models for human microbiome studies and immune response research in the lung. Organs-on-chip provide an attractive alternative as they allow the use of human immune cells and defined microbial communities up to complex human microbiota samples to study mechanisms of human respiratory infection under defined and reproducible conditions.

**Figure 1.**
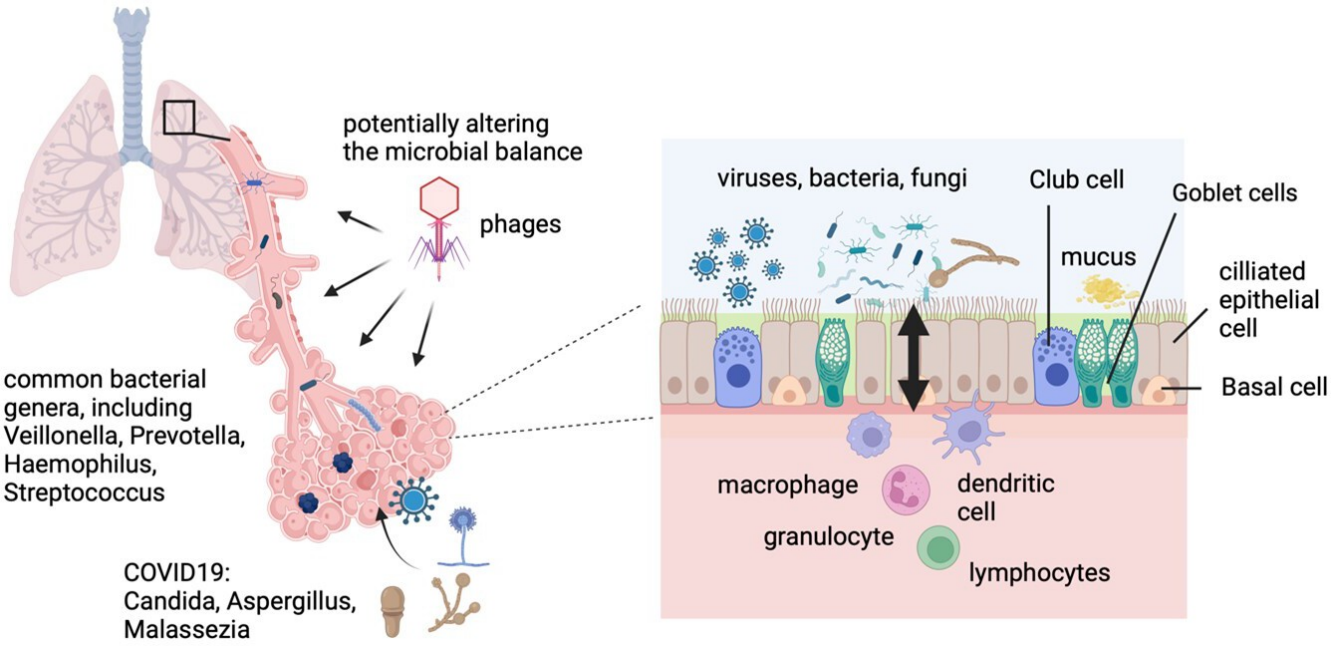
The lung microbiome plays a crucial role in maintaining respiratory health and immune function. It is composed of various bacterial genera such as *Veillonella*, *Prevotella*, *Haemophilus* and *Streptococcus*, which form the core of this microbial community. The balance of this microbiome can be disrupted by viruses and bacteriophages, leading to respiratory health issues. Additionally, common fungi like *Candida*, *Aspergillus* and *Malassezia* species can cause secondary infections in COVID-19 patients. The lung microbiome also contributes to immune development and interacts with the host immune system to maintain lung homeostasis (created with Biorender.com).

Most of the microbiome in the lungs is composed of bacteria. Individuals may differ in their bacterial community, but common genera are *Veillonella*, *Prevotella*, *Haemophilus* and *Streptococcus* [70]. The lung can further harbour various viruses, including bacteriophages. These viruses can interact with bacteria and change the balance of the microbial community. Fungi also colonize the lungs. *Candida, Aspergillus* and *Malassezia* species are common fungi in the lung microbiome, particularly in patients suffering from COVID-19 [71–73]. Like the gut microbiome, the lung microbiome contributes to metabolic processes and can metabolize compounds such as mucins and xenobiotics [74]. Importantly, the lung microbiome further plays a major role in immune development and regulation and contributes to homeostasis within the lung [75–77] (figure 1).

## 3. The gut–lung axis: a two-way street

Respiratory viruses like influenza, RSV and SARS-CoV-2 mainly affect the respiratory system, but they can also impact the gut by affecting the microbial communities and the immune system [78–80]. The concept of the gut–lung axis represents a fascinating paradigm that delineates the bidirectional communication between the gastrointestinal tract and the lungs [31,81]. Disruptions or imbalances in the gut, especially concerning its diverse microbiota, can be mirrored in the lung through this axis, and vice versa [76,82,83]. Given the increasing prevalence of gastrointestinal and respiratory disorders worldwide, deepening our understanding of communication along the gut–lung axis is crucial to decrease disease susceptibility and improve viral clearance.

**Figure 2.**
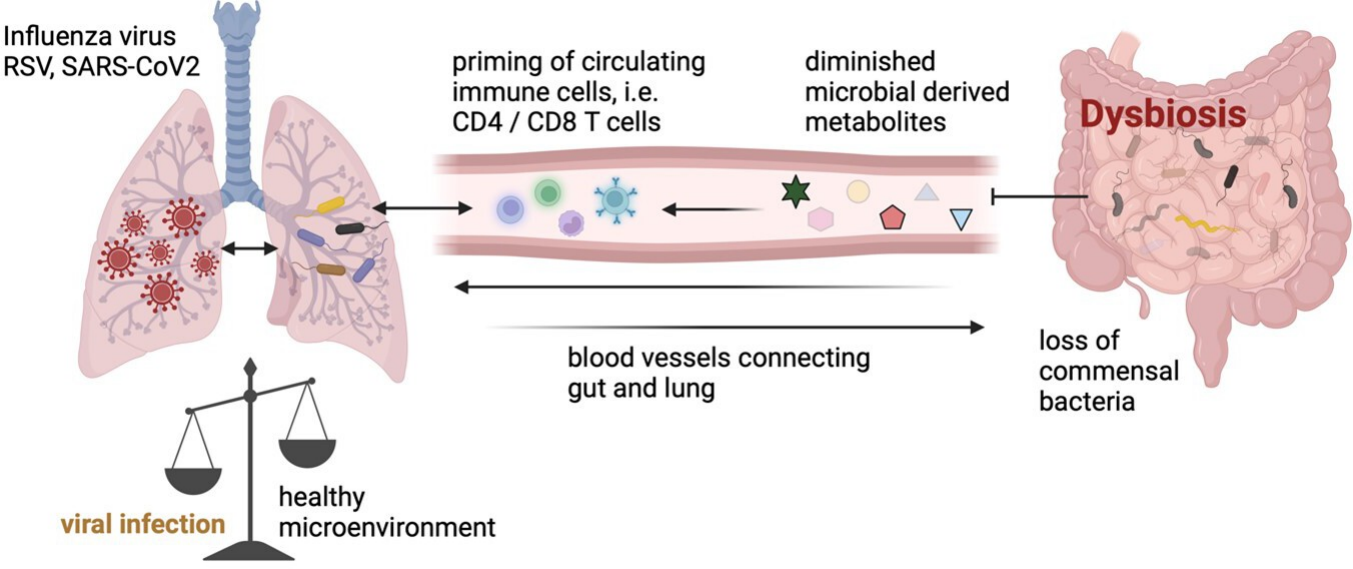
The gut–lung axis mediates the bidirectional communication between the gastrointestinal and respiratory systems. The gut microbiota has a critical role in immune modulation, including activating the inflammasome and priming T cells. An unbalanced gut microbiome can compromise gut barrier function, leading to systemic inflammation, alterations in the formation of microbial metabolites and dysregulation of the immune response, thereby influencing the severity of viral respiratory infections (created with Biorender.com).

Understanding the complex interplay between viral respiratory infections and the gut–lung axis provides insight into the systemic impacts of microbial imbalances (figure 2). It has been observed that influenza viruses and SARS-CoV-2 can alter the gut microbiota composition, even in the absence of gastrointestinal symptoms [79,82,84]. This could result in the migration of microbes and microbial components into the bloodstream, eventually leading to inflammation and systemic infections. Conversely, a healthy gut microbiota can modulate the immune response in the lungs, potentially reducing the severity of influenza infections [85].

It has been demonstrated that the commensal microbiota plays a critical role in regulating immune responses to respiratory influenza virus infection by influencing the generation of virus-specific CD4 and CD8 T cells and antibody responses. Ichinohe *et al*. [86] conducted a study that showed how neomycin-sensitive bacteria play a crucial role in triggering a productive immune response in the lungs. They found that antibiotics can weaken the immune system, which can be mitigated by administering Toll-like receptor ligands locally or distally [86]. This study emphasizes the important role of lung microbiota in activating the inflammasome and priming T cells in the respiratory mucosa. Recent research also suggests RSV infections can alter gut microbiota composition, particularly in infants [80]. The altered microbiota can impact the maturation of the immune system, potentially influencing the severity and long-term implications of RSV infections [87]. Further exploration is needed to determine if pre-existing gut microbial imbalances increase susceptibility to severe RSV manifestations. Severe COVID-19 patients were found to have elevated antibody levels specific for the yeast *Candida albicans* and showed parallel intestinal *Candida* overgrowth and elevated circulating neutrophil numbers. Experimentally, this was modelled by colonizing mice with the patient isolates, which then also developed pulmonary neutrophilia. This study crucially linked intestinal mycobiota dysbiosis with the activation of neutrophil progenitors and COVID-19 severity [88].

### 3.1. Chronic respiratory diseases linked to viral infections

It has become evident that microbial shifts influence our response to infections and play a pivotal role in chronic respiratory conditions like asthma and chronic obstructive pulmonary disease (COPD) [89–92]. Asthma, marked by airway inflammation and hyper-responsiveness, frequently worsens in the presence of respiratory viruses such as rhinovirus, RSV and influenza, often leading to acute symptom exacerbation and respiratory distress necessitating medical intervention [93]. In addition, asthma has also been linked to dysbiosis in the gut microbiome [94]. Microbial metabolism by-products like SCFAs can modulate immune responses, and imbalances in these metabolites can impact asthma severity [95]. This may affect the regulatory T-cell populations and other immune components, skewing the immune system towards an allergic phenotype. Notably, intestinal colonization by *C. albicans* can induce Th17 cells, contributing to allergic airway inflammation, with intestinal CX3CR1+phagocytes playing a crucial role in fungal recognition and driving lung inflammation [96–98,99]. However, it is important to note that there remains considerable skepticism regarding these observations, as it is challenging to determine whether changes in the microbiota are a cause or a consequence of asthma. The causality between microbiota alterations and asthma thus remains a subject of ongoing debate and requires further investigation.

**Figure 3.**
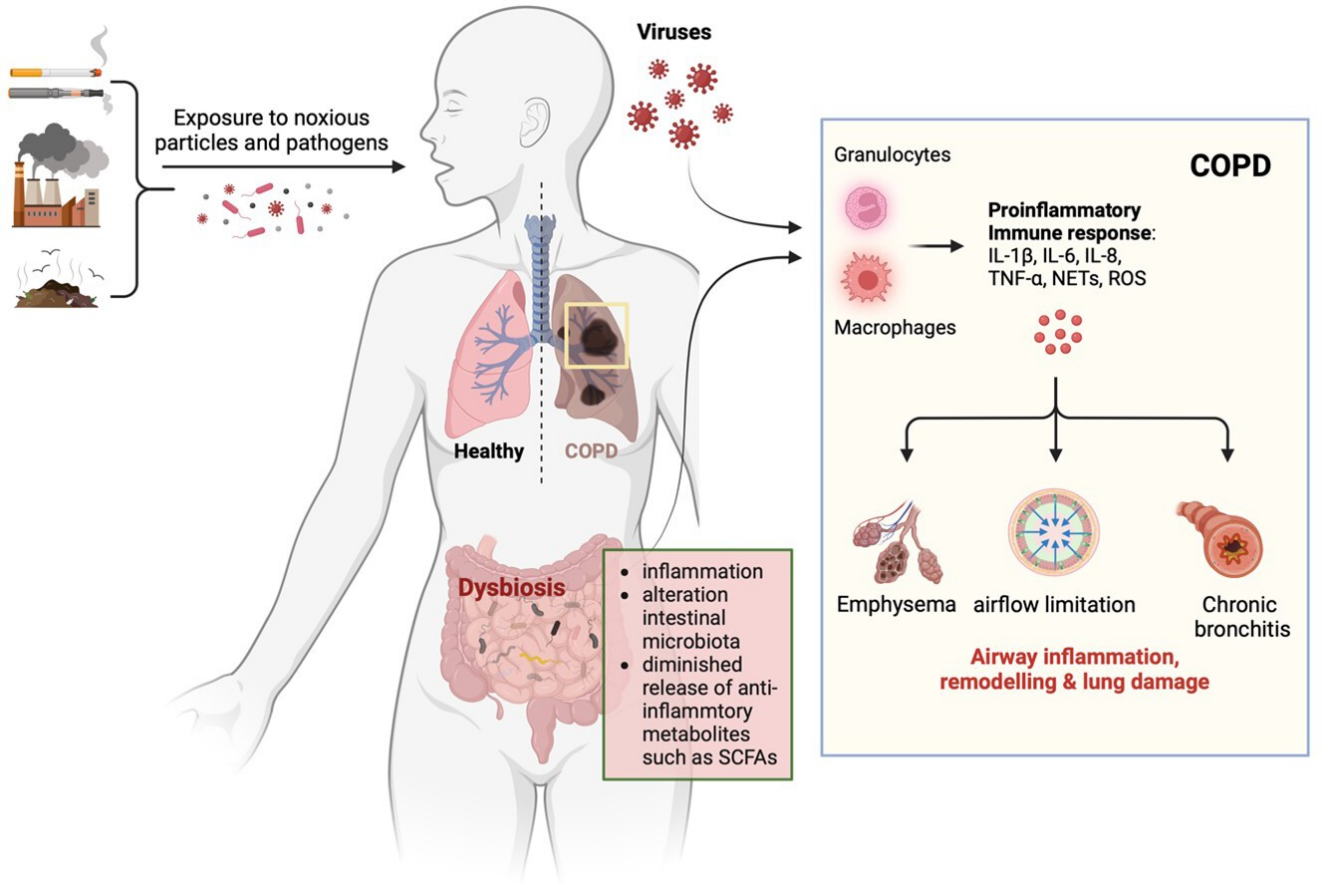
Exposure to cigarette smoke, air pollution and pathogens can induce chronic obstructive pulmonary disease (COPD). COPD patients exhibit heightened susceptibility to respiratory viral infections due to underlying chronic inflammation, exacerbated by dysbiosis-associated inflammation and diminished anti-inflammatory microbial metabolites, such as short-chain fatty acids (SCFAs), produced by commensal intestinal microorganisms (created with Biorender.com).

COPD, characterized by chronic bronchitis and emphysema, leads to airflow limitation and respiratory distress. While smoking is the primary risk factor, gut microbiota alterations are also associated with disease progression. Patients with COPD often show decreased intestinal bacterial diversity and increased species frequencies [100]. These changes could potentially lead to a more pronounced systemic inflammatory response capable of exacerbating lung inflammation in COPD [89] (figure 3). Individuals with COPD face heightened concerns with influenza infections, as they tend to experience more severe symptoms and prolonged recovery periods [101]. Chronic inflammation in COPD can be intensified by influenza, leading to acute exacerbations, hospitalizations or increased mortality [102]. For example, the overgrowth of certain gut bacteria like Enterobacteriaceae has been correlated with frequent COPD exacerbations [101]. The presence of intestinal dysbiosis may increase susceptibility to severe viral infections. Consequently, dysbiosis could potentially escalate the severity of respiratory viral infections or the frequency of COPD or asthma exacerbations [31].

### 3.2. Orchestration of the immune response along the gut–lung axis

The gut is home to most human immune cells, particularly within the gut-associated lymphoid tissue (GALT). Here, commensal bacteria interact directly and indirectly with immune cells, driving the differentiation of T cells into various subsets, including regulatory T cells (T_reg_) and effector T cells (T_eff_), thereby influencing systemic immunity [103]. T_reg_ cells are a specialized subset of T cells that suppress the activation and effector function of other immune cells, preventing autoimmunity. They maintain immune self-tolerance by inhibiting the immune system from attacking self-tissues and maintaining tolerance to self-antigens. T_eff_ cells, on the other hand, are the immune system’s primary response cells that act directly against infections. These cells include various subsets, such as T_H_1, T_H_2, T_H_17 and cytotoxic T lymphocytes (CTLs), each with specific roles in defending against different pathogens and contributing to the immune response [104].

Intestinal dysbiosis has been increasingly linked to heightened severity of respiratory infections [105–107]. Several mechanisms underlie this connection: gut dysbiosis can lead to a compromised mucosal barrier, allowing bacterial translocation and triggering systemic inflammation. This systemic inflammatory state can precondition the lungs to be more vulnerable to pathogens, disrupting the lung’s innate immune responses [31]. Dysbiosis can also shift the balance of T_reg_ versus T_eff_ cells, skewing immune responses towards either hyper-responsiveness or suppression, neither ideal for efficiently combatting respiratory pathogens [108,109]. A recent study revealed *C. albicans* as an inducer of Th17 cells, highlighting a connection between protective Th17 responses to *C. albicans* in the gut and potential risk for pulmonary inflammatory diseases [96].

In addition, microbial imbalances can alter the production of microbiota-derived metabolites, such as SCFAs and tryptophan metabolites. As these metabolites play crucial roles in modulating immune responses, their alteration can directly impact lung immune homeostasis, affecting the respiratory system’s capacity to fend off infections.

### 3.3. SCFAs bridge the gut microbiota and respiratory immunity

The composition of the gut microbiota determines the ability to ferment dietary fibres, producing consequential metabolites such as SCFAs [95]. Among these, butyrate, propionate and acetate stand out in their prevalence and significance. Produced predominantly in the colon, these SCFAs are absorbed into the bloodstream, exerting systemic effects on distant organs, with the lungs being a notable beneficiary. Butyrate is a well-known anti-inflammatory microbial-derived compound that inhibits the production of inflammatory mediators like TNF-α and IL-6 by innate immune cells such as macrophages and dendritic cells [110]. Beyond simply modulating inflammation, SCFAs also play an important role in modulating T-cell activity. They are instrumental in guiding the differentiation of T_reg_ within the gut, a process vital for preserving immune equilibrium. This fine-tuning of the T-cell response can act as a buffer, limiting excessive immune reactions in the lungs and potentially protecting against inflammatory lung disorders, including asthma [111]. Propionate, another SCFA, has been pinpointed for its capacity to reduce the release of pro-inflammatory compounds by neutrophils, presenting a mechanism to attenuate airway inflammation [112]. Furthermore, the epithelial integrity of the gut, which forms a frontline defence against pathogenic translocations, is bolstered by SCFAs, primarily by butyrate [113]. The various roles played by SCFAs emphasize their significance in the connection between gut health and lung immune responses.

Recent studies indicate that SCFAs can significantly impact lung immune responses. Cait *et al*. [99] found that SCFAs butyrate, propionate and acetate play a key role in alleviating allergic lung inflammation by modulating the immune response of dendritic cells and T cells, particularly by promoting regulatory T-cell differentiation while reducing T helper type 2 (T_H_2) cell activity. The T_H_2 subset of CD4^+^ T cells plays a crucial role in the immune response to respiratory infections by promoting antibody production, mucus secretion and eosinophil recruitment. They are primarily involved in responses against extracellular pathogens and drive the pathophysiology of allergic diseases, including asthma, by secreting cytokines like IL-4, IL-5 and IL-13. These cytokines help orchestrate a type 2 immune response, which can exacerbate inflammation and airway hyper-responsiveness in the context of respiratory infections [114]. SCFAs notably downregulated pro-inflammatory cytokines and upregulated anti-inflammatory cytokines, leading to a marked decrease in allergic responses in the lungs [99]. Further, the study by Trompette *et al*. highlights the impact of SCFAs on lung health and their potential role in viral infections [115]. Dietary fibre influences the gut microbiota composition [116]. This change leads to increased production of SCFAs like propionate, which enter the circulation and impact lung immunity. Mice on a high-fibre diet with higher circulating SCFA levels showed reduced allergic lung inflammation. This effect is linked to changes in bone marrow haematopoiesis and the recruitment of dendritic cells (DCs) to the lungs. These DCs exhibit high phagocytic capacity but a reduced ability to promote T_H_2 cell effector functions, suggesting a shift towards a more anti-inflammatory or balanced immune response in the lungs [112]. Given the critical role of immune response modulation in viral infections, these findings imply that dietary SCFAs might influence the severity of viral lung diseases by shaping the pulmonary immune environment. SCFAs further promote the release of the anti-inflammatory cytokine IL-10, supporting the mitigation of lung inflammation and tissue damage typically seen in severe influenza A infections [112].

Epigenetic modifications triggered by gut and lung microbiota play a crucial role in preparing the innate immune system to identify infected cells better and initiate an effective immune response. Recent studies have shed light on the significance of DNA methylation, histone modification and non-coding RNAs in regulating gene expression in immune cells interacting with microbial communities [95]. These alterations in gene expression can lead to changes in cytokine production, improved phagocytosis and better elimination of pathogens, thus strengthening the host’s defence mechanisms against infections.

Influenza A virus infections often lead to secondary bacterial infections, which can be exacerbated by disruptions in the gut microbiota. Studies have shown that influenza infections in mice can cause temporary changes in the gut microbiota’s composition and fermentative activity due to reduced food intake. These changes can negatively impact defence against respiratory bacterial superinfection, such as pneumococcal infection. It was found that reduced production of SCFA acetate during influenza virus A infection diminishes the bactericidal activity of alveolar macrophages. Supplementing acetate in mice significantly decreased bacterial loads and was protective against bacterial superinfection. This protective effect was demonstrated to depend on the activation of free fatty acid receptor 2 (FFAR2), which reduced bacterial loads and led to higher survival rates in mice [117]. SCFAs further mediate protection against viral respiratory infection by their ability to modulate immune responses, particularly through enhancing type I interferons (IFNs), crucial in initial respiratory defence.

Gut microbiota-derived acetate shows promise in protecting against respiratory syncytial virus (RSV) infection. *In vitro* studies revealed that acetate treatment of human pulmonary epithelial cells reduces RSV infection and boosts the expression of key antiviral genes like RIG-I and ISG15. This protective effect was also observed in a mouse model, where intranasal acetate treatment led to faster recovery, reduced viral load, and enhanced expression of interferon-β and RIG-I in lung tissue. Furthermore, the severity of bronchiolitis in infants is linked to their gut microbial profile and faecal acetate levels. Higher fecal acetate concentrations were associated with less severe clinical symptoms, such as higher oxygen saturation and shorter fever duration [118]. *Ex vivo* treatment of respiratory epithelial cells from RSV-infected patients with acetate reduced viral load and increased expression of antiviral genes. However, the beneficial effects of acetate were not observed in cells from SARS-CoV-2 infected patients, indicating a virus-specific protective mechanism [118]. In neonatal mice, probiotics showed significant therapeutic effects against RSV-induced lung pathology. The probiotic mixture, containing *Lactobacillus rhamnosus* GG, *Escherichia coli* Nissle 1917 and VSL#3, was administered either 1 week before or concurrently with RSV infection. This treatment protects against RSV by enhancing the antiviral response, particularly through IFN-β production by alveolar macrophages (AMs). Additionally, the probiotics reversed gut dysbiosis in RSV-infected mice, increasing the abundance of SCFA-producing bacteria. This shift resulted in elevated serum SCFA levels, particularly acetate, which was linked to enhanced IFN-β production in AMs [119]. A drop in SCFA serum levels was also observed in the Syrian hamster model infected with SARS-CoV-2, which led to changes in the gut microbiota composition, characterized by a decrease in SCFA-producing bacteria belonging to the *Ruminococcaceae* and *Lachnospiraceae* families. Although SCFA supplementation during the infection did not significantly impact clinical outcomes or inflammation in this model [120], the supplementation with acetate showed beneficial effects in combinatory antiviral treatment against SARS-CoV-2 [121]. The fatty acid α-linolenic acid (ALA) was shown to interact with and degrade viral particles, including SARS-CoV-2. Combining ALA with acetate in liposomes significantly inhibited viral activity, including reducing viral plaque formation and the release of pro-inflammatory cytokines IL-6 and IL-1β, while boosting IFN-β expression [121].

Current research thus focuses on the potential use of probiotic strains to protect from severe respiratory infections. In this context, the protective effect of *Lactobacillus mucosae* against RSV infection was recently studied in a mouse model. Oral administration of three *L. mucosae* strains to young mice inhibited RSV replication, reduced inflammatory cell proportions in the blood and differentially regulated immune responses, including increased levels of key cytokines like IFN-β and TNF-α. Notably, these strains modulated the gut microbiota composition and enhanced the SCFA levels [122]. Further, the probiotic strain *Lactobacillus plantarum* has shown potential in modulating immune responses against both COVID-19 and influenza. In patients suffering from COVID-19, *L. plantarum* significantly increased the innate cytokine index, indicating robust immunomodulatory ability. This was observed through a notable decrease in IL-6 levels and enhancement of natural killer cell activity, suggesting its efficacy in mimicking early immune responses to viral infection and potentially preventing COVID-19 [123].

For influenza, a study showed that elderly who consumed *L. plantarum* post-influenza vaccination had increased levels of influenza-specific IgA and IgG antibodies, indicating a potential immunostimulatory effect associated with *L. plantarum* uptake [124]. In other studies involving elderly individuals consuming a probiotic dairy drink containing *Lactobacillus casei* DN-114001, an enhanced antibody response to influenza vaccination was also reported. This was evident in increased antibody titres against various vaccine strains, highlighting the potential of immune response boosting in the elderly population [125]. However, a study examining *Lactobacillus casei* 431 in healthy adults showed no significant impact on the response to influenza vaccination but did demonstrate a reduction in the duration of upper respiratory symptoms [126].

### 3.4. Tryptophan metabolites and biliary acids in the crosstalk of gut and lung

The relevance of the gut microbiota for respiratory infections is further demonstrated by tryptophan metabolites formed in the intestine that modulate the immune response in the lung. Tryptophan is an essential amino acid obtained through diet and can be metabolized by the gut microbiota into several bioactive metabolites. One of the best-studied pathways involves the transformation of tryptophan into indole derivatives, including indole-3-aldehyde, indole-3-acetic acid and indole-3-propionic acid [127]. These metabolites have been shown to modulate mucosal barrier functions and influence immune cell behaviour, particularly within the gut [128–130]. However, their impact extends beyond the intestinal environment. For respiratory health, emerging evidence suggests that tryptophan metabolites can play a role in modulating lung inflammation [128,131]. For instance, tryptophan metabolites have been shown to act on the aryl hydrocarbon receptor (AhR), a ligand-activated transcription factor pivotal in immune regulation [128]. Activation of AhR in respiratory tissues can mediate anti-inflammatory effects, and disruptions in tryptophan metabolism with subsequent alterations in its metabolite profile are linked to imbalances in immune responses, contributing to exaggerated inflammatory reactions in the lung [132].

**Figure 4.**
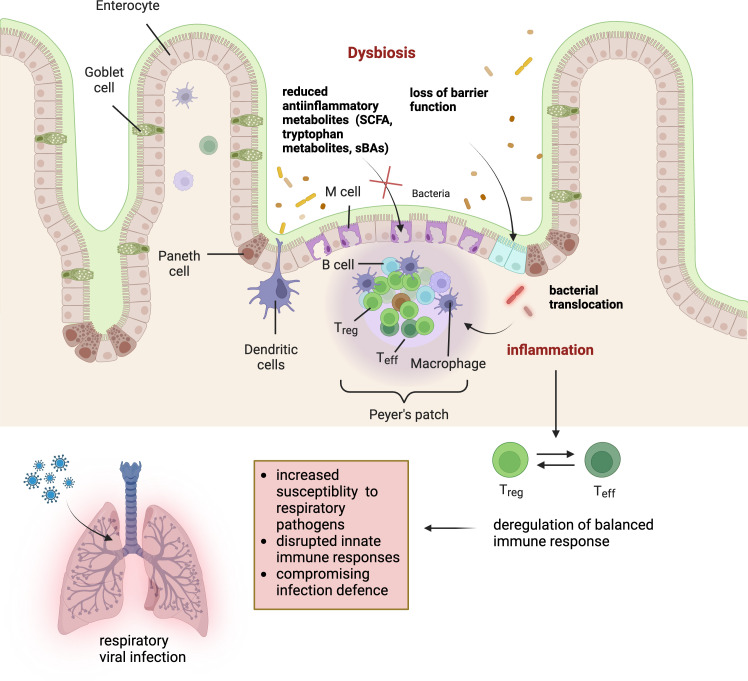
Links between the gut microbiome and susceptibility to respiratory infections. Excess opportunistic pathogens and a reduced abundance of beneficial microorganisms undermine the gut barrier function, enabling bacterial translocation, which induces a shift in the immune balance within the gut-associated lymphoid tissue (GALT), favouring a pro-inflammatory immune response. In addition, the loss of anti-inflammatory microbial metabolites, including short-chain fatty acids (SCFAs), tryptophan metabolites and secondary bile acids (sBAs), which play crucial roles in maintaining immune regulation and gut integrity, further drives a systemic inflammatory response. This pro-inflammatory milieu conditions the lungs to a heightened vulnerability, impairing the innate immune response and compromising the defence against viral pathogens (created with Biorender.com).

Furthermore, bile acids (BAs) are recognized for their role in dietary lipid absorption in the intestine. Recently these metabolites emerged as significant signalling molecules mediatfing the crosstalk between the gut and distant organs, including the lungs [133]. Synthesized in the liver from cholesterol, BAs undergo biotransformation by the gut microbiota, producing secondary bile acids, which can exert systemic effects. The discovery that BAs can affect the immune system in the lungs provides new insights into the complexities of the gut–lung axis. A recent study investigated the relationship between cystic fibrosis (CF) and intestinal microbiota-related bile acids (BAs). CF is a disease that causes thick and sticky mucus to build up, leading to blockages, infections and scarring of the respiratory epithelia. The study found a significant association between BAs and airway inflammation in CF patients. Detection of BAs in the bronchoalveolar lavage fluid (BALF) of infants with CF was found to be strongly associated with markers of airway inflammation, more frequent exacerbations and distinct microbial profiles in the lungs [134]. Receptors such as the farnesoid X receptor (FXR) and the G protein-coupled bile acid receptor (TGR5) are known targets of bile acids [135]. Their activation can influence immune responses, inflammation and metabolic processes. In the context of the lungs, alterations in BA composition and signalling have been linked to various respiratory conditions. Several underlying mechanisms can intensify lung inflammation due to dysregulation in BA homeostasis. BAs have been demonstrated to cause lung damage and inflammation in cultured lung epithelial cells by repressing hypoxia-inducible factor 1 [136]. Furthermore, the increased levels BAs in the lungs have been linked to compromised innate immune function [137], decreased levels of crucial pulmonary surfactants [138] and induction of lung fibrosis [139]. However, in another study in T_reg_-deficient scurfy (SF) mice suffering from dysbiosis in the gut microbiota, treatment with different antibiotics restored levels of primary and secondary BAs, which was found to be associated with reduced lung inflammation and prolonged lifespan of mice by lowering intestinal IL-6 release [140]. The disruption of BA receptors or alterations in BA metabolism can potentially lead to exaggerated lung inflammatory responses via the modulation of macrophage functions. Perino *et al*. demonstrated that activation of the BA-receptor TGR5 in macrophages suppresses inflammation by inhibiting chemokine expression mediated through the AKT-mTOR signalling pathway. This pathway induces differential translation of the C/EBPβ isoform liver inhibitory protein, which acts as a dominant-negative regulator, thereby reducing chemokine expression. These findings suggest that alterations in BA receptor signalling, specifically TGR5, can influence macrophage-mediated inflammatory responses, which could be relevant to lung inflammation and immune modulation [141]. Moreover, certain respiratory infections, such as those caused by influenza, might further disturb BA homeostasis, creating a feedback loop that influences both gut homeostasis and respiratory function [142]. A summary of mechanisms and concepts on how dysbiosis relates to susceptibility to respiratory viral infections is provided in figure 4.

However, while interactions between the gut and lung microbiota in the context of respiratory viral infections are increasingly recognized, many underlying mechanisms remain unclear. This uncertainty raises important questions. How exactly do microbial changes in the gut influence immune responses in the lungs, and do microbial metabolites primarily drive these effects, or are other mechanisms involved? Further, what role do specific microbial species play in modulating susceptibility to viral infections, and how can this knowledge be harnessed to improve treatment outcomes?

The correlations between alterations in gut microbiota and respiratory viral infections have been documented, although establishing causality is often challenging. The complex interaction between the gut and lung microbiomes in the context of respiratory viral infections, such as influenza, RSV and SARS-CoV-2, presents both challenges and opportunities for advancing medical research and clinical practice. The emerging understanding of the gut–lung axis underscores the necessity for interdisciplinary approaches to investigate the systemic effects of microbial imbalances and their implications for respiratory health. One key area for future research is the identification of specific microbial species or metabolites that play critical roles in modulating immune responses during respiratory infections. Understanding which elements of the gut microbiota are most influential in either exacerbating or mitigating respiratory illnesses could lead to targeted probiotic or prebiotic therapies. Such therapies could be designed to restore or maintain a healthy gut microbiota, thereby improving lung health and reducing the severity of viral infections. Additionally, the role of diet and nutrition in shaping the gut microbiota and, by extension, the gut–lung axis, warrants further investigation. Dietary interventions that promote the production of beneficial microbial metabolites, such as SCFAs, could offer a non-pharmacological approach to enhancing immune responses to respiratory infections. This approach could be particularly valuable in populations at higher risk for severe respiratory diseases, such as the elderly or those with chronic conditions. The potential for gut microbiota modulation to influence vaccine efficacy is another promising avenue of research. However, due to the biased design of available studies, conclusions remain controversial [143]. Understanding these relationships could, however, lead to new strategies that optimize vaccine responses through dietary or microbial interventions, thereby improving public health outcomes during pandemics or seasonal outbreaks. Another consideration is the variability in microbiome composition between individuals, which depends on factors such as age, geography, diet and lifestyle that can lead to significant differences in the gut and lung microbiota, making it challenging to generalize findings across populations. This variability underscores the need for personalized research and clinical practice approaches.

However, the bidirectional nature of the gut–lung axis adds complexity, as changes in one organ system can influence and be influenced by changes in the other. Thus, while the gut–lung axis is a promising area of research, many questions remain unanswered, particularly regarding the precise mechanisms through which this axis influences respiratory health and disease. Moreover, as microbiome research continues to expand, there is a growing need for robust, human-relevant models to replicate defined, although not fully complete, functional units of the complex interactions between different organ systems.

To address these open questions and challenges in the experimental approach, OoC technology offers promising solutions. OoC models allow the recreation of human tissue microenvironments under controlled conditions, enabling the study of complex inter-organ interactions, which is not possible with conventional *in vitro* or animal models. By incorporating human immune cells and defined microbial communities, OoC systems can provide a more accurate representation of functional organ units and defined aspects of human physiology, particularly in the context of the gut–lung axis.

Though organ-on-chip models offer many advantages, they also have limitations. OoC systems do not fully capture the complexity of whole-organism interactions or the influence of the broader physiological environment. However, integrating data from OoC models with findings from animal studies, clinical trials and epidemiological research will be a valuable approach to gaining a comprehensive understanding of how the microbiota determines the course and severity of respiratory infections.

## 4. Combining OoC platforms and *in silico* analysis

Combining OoC platforms with *in silico* analysis enhances the reliability of microbiome research. OoCs provide standardized human models with reproducible conditions that closely match physiological conditions in the human host. This integration allows for precise hypothesis generation and validation, improving the understanding of how microbiome shifts influence disease susceptibility and treatment effectiveness.

Statistical analyses link microbiome features with infection states. Discriminatory methods, including machine learning, identify features that differentiate groups (e.g. infected versus healthy). Though sensitive to cohort definitions, these methods have linked bacterial taxa with COVID-19 progression and severity [144,145]. Experimental validation is thus crucial to avoid spurious conclusions [146].

**Figure 5.**
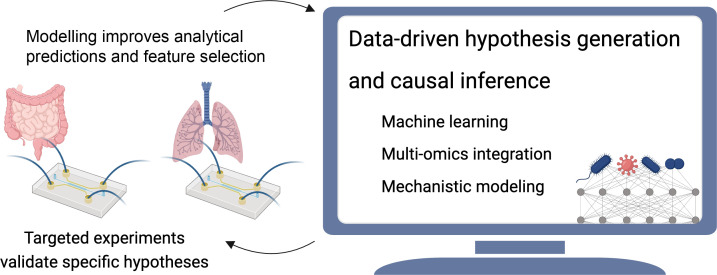
Integrating microbiome data analyses with targeted experiments in OoC offers an opportunity to understand how the human microbiome influences infection susceptibility and severity. Microbiome data analysis (e.g. machine learning or mechanistic modelling) helps to define hypotheses about the effects of specific microbial strains or microbially derived metabolites (e.g. SCFAs), which can be validated in OoCs. Experimental data can be used to computationally identify which microbial and host features (e.g. microbial species, gene expression) interact in response to viral infections (created with Biorender.com).

Mechanistic modelling, such as flux-balance analysis, can help identify causal links between the microbiome, immune system and viral infections [147–149] and allows for the revelation of metabolic networks and the prioritization of hypotheses for validation. Recent approaches have linked inflammatory diseases to bacterial metabolites, improving predictions with experimental data [150,151]. In this context, OoCs are ideal systems for generating such data in biologically relevant contexts. Technological advances for generating high-throughput omics data, a burgeoning bioinformatics field and increasingly biologically diversified OoC platforms offer an exciting opportunity to understand the mechanisms underlying links between microbiome, infection and immunity (figure 5).

In this context, *in silico* analysis combined with OoC can also revolutionize the study of zoonotic infections by enabling the analysis of biological data from multiple species and facilitating the understanding of zoonotic diseases at a granular level. OoC technology facilitates studies encompassing both human and animal cells, which can significantly broaden research possibilities beyond the constraints of species-specific models. This flexibility is essential to investigate infection with zoonotic pathogens. OoC models represent potent tools for studying interspecies disease transmission and the evolution of pathogens, which are fundamental aspects of emerging infectious diseases and zoonosis. These models enable replicating human and animal physiological conditions, allowing researchers to observe how pathogens like viruses and bacteria interact with cells from different species. Comparative studies between human and animal models are crucial in understanding host responses and adaptability mechanisms of zoonotic pathogens. The advantage of using a standardized platform suitable for culturing tissues of different hosts is that one can compare how infection of the same sample progresses in distinct environments under similar conditions. This approach will provide insights that are crucial for developing strategies to predict and mitigate the impact of future zoonotic outbreaks.

Artificial intelligence (AI) and image analysis algorithms can be trained to recognize overarching patterns in cell behavior, predict outcomes of pathogen interactions and optimize the conditions within the highly standardized OoC systems for more accurate disease modelling. Through machine learning, AI can help identify novel biomarkers for diagnostics, uncover therapeutic targets and test the efficacy of novel drug candidates, even in a personalized approach. This can lead to developing predictive models for disease spread and treatment responses, informing the design of more effective therapeutic strategies. This approach can help monitor the evolution of infections, providing an early warning system for potential outbreaks.

## 5. OoC models: recreating the respiratory and intestinal microenvironment

By connecting organ models of the lung and gut, researchers can explore the systemic interactions between these organs, including the role of gut microbiota in respiratory infections. Interconnected multi-organ systems will enable the investigation of how alterations in the gut environment, such as dysbiosis, alter the response of the lung to infections and vice versa, potentially offering new insights into potential holistic treatment approaches for respiratory viral infections. Applying OoC in modelling the gut–lung axis could allow more controlled and detailed studies of the complex inter-organ communications and their mechanistic implications in the context of respiratory infections. As complex *in vitro* systems OoC offer a scalable biological complexity in its cellular composition and arrangement tailored to individual study needs. These models could contribute to bridging the gap between traditional *in vitro* studies and *in vivo* human responses. With its ability to monitor infection and drug interactions with human tissues in real-time, these models could provide additional information that allows for a deeper comprehension of the pharmacokinetics and pharmacodynamics of new substances and the pathophysiology of respiratory diseases. Thus, lung- and gut-on-chip models can be valuable tools for identifying possible side effects early in drug development and help in refining therapeutic approaches (figure 5).

### 5.1. Basics of OoC technology

The evolution of OoC technology marks a significant leap in biomedical research, tracing its origins from rudimentary *in vitro* models to the current sophisticated systems that mimic defined functional units of human organs *in vitro*.

Initially, the research relied on simplistic two-dimensional cell cultures, which, while useful, fell short in replicating the complex spatial architecture and dynamic microenvironment of human tissues. This gap led to the advent of OoC technology, a transformative progression aiming to bridge the divide between *in vitro* studies and *in vivo* conditions. This advancement was driven by the need to overcome the limitations inherent in traditional two-dimensional (2D) cultures, such as the inability to accurately model cell–cell and cell–extracellular matrix interactions, which are crucial for understanding tissue physiology and pathology. Therefore, the development of OoC technology responded to an imperative demand in medical research for more physiologically relevant models that could provide deeper insights into human biology, disease mechanisms and drug responses, ultimately leading to more effective therapeutic interventions and a reduction in reliance on animal testing.

With the utilization of microfluidic perfusion, these systems allow a three-dimensional (3D) cell culture with precise control of biochemical and biophysical conditions, enabling multiple cell types to form tissue with defined organotypic functionality. The European Organ-on-Chip Society (EUROCS) defines OoC as a ‘fit-for-purpose microfluidic device, containing living engineered organ substructures in a controlled microenvironment, that recapitulates one or more aspects of the organ’s dynamics, functionality and (patho)physiological response *in vivo* under real-time monitoring’ [152]. Microfluidic biochips that are used for OoC technology consist of tiny channels and compartments that allow for precise delivery of nutrients and growth factors to cells while removing waste products. OoCs play a critical role in improving the accuracy of drug efficacy and toxicity assessments, pathophysiological studies and personalized medicine approaches [153,154]. They effectively bridge the gap between traditional cell culture methods and animal models.

Most OoC systems contain a membrane that mimics an extracellular matrix as a cell and tissue scaffold. The natural ECM contains different proteins, glycoproteins and proteoglycans that provide the physical, biochemical and biomechanical properties of the physiological microenvironment that determines crucial functions in cell adhesion, proliferation, differentiation, morphology and even migration [155,156].

### 5.2. Lung-on-chip models

OoC technology, particularly lung-on-chip models, provides an elegant approach to studying viral respiratory infections by mimicking the complex pulmonary tissue architecture and dynamic mechanical forces. These models recreate the essential features of the respiratory tract, including the alveolar–capillary interface for gas exchange and barrier function studies [157–159] and large airway environments for researching respiratory diseases like asthma and chronic obstructive pulmonary disease (COPD) [160]. Some lung-on-chip models incorporate flexible membranes that simulate breathing by mechanically stretching to replicate lung expansion and contraction [157,161,162]. This capability is crucial for exploring lung physiology, disease progression and drug response in a physiologically relevant manner. By accurately replicating *in vivo* conditions, lung-on-chip technology enhances the reliability of respiratory research, offering profound insights into tissue responses to pathogens and pharmacological agents [163,164].

A flexible cell substrate is required to recreate breathing mechanics, so most of the lung-on-chip membranes that support mechanical strain are made of polydimethylsiloxane (PDMS). However, this material does not reflect the natural biochemical and biophysical characteristics of the extracellular matrix (ECM). Further, non-selective absorption of hydrophobic compounds by the PDMS membrane potentially contributes to bias in drug testing studies [165].

Mechanical strain applied to cell culture substrates that mimics the regular stretching and relaxation experienced by lung tissues *in vivo* under physiological conditions is crucial in achieving biological relevance within lung-on-chip systems. Mechanostimulation of lung cells is critical for promoting pulmonary branching and alveolar differentiation during normal lung development [166,167]. However, these mechanical forces also contribute to various lung diseases, including acute lung damage, pulmonary oedema and pulmonary fibrosis [168]. Breathing mechanisms shape the physical microenvironment of the respiratory tract and influence immune responses. A study by Stucki *et al*. [169] demonstrated that primary human pulmonary alveolar epithelial cells cultivated with mechanical strain showed considerably increased metabolic activity with increased IL-8 release compared with static culture.

Bai *et al*. [170] showed moderate production of the S100A7 protein by both alveolar epithelium and endothelium during applying breathing motions. This protein binds to a receptor for advanced glycation end products (RAGE), triggering an innate immune response that provides protection against viral infections. In the absence of respiratory motions, however, S100A7 levels drop, thereby increasing the susceptibility to viral infections.

Air–liquid interface (ALI) refers to conditions frequently used in cultured airway epithelial cells [171]. The critical characteristic of ALI culture is that the cells’ basal side receives nourishment from contact with liquid cell culture media while their apical surface is exposed to air [171]. Compared with traditional and submerged cultures, this arrangement facilitates cell development towards a mucociliary phenotype, mimicking *in vivo* settings more closely. By exposing the cells to air, more oxygen is supplied to the cells, which further causes increased cellular respiration and a transition to a more aerobic state, leading to an acceleration of oxidative cellular metabolism and as already stated, promoting cellular differentiation with improved tight junction formation [172]. Further, at ALI culture, medications delivered as aerosols or particles do not dilute or undergo structural changes when interacting with cell culture medium, as opposed to cultures involving direct interaction with the medium. Overall, applying ALI to airway epithelial models improves their physiological properties and allows a more precise study to the different pathogenic conditions [171].

These systems are crucial for studying the interactions between respiratory cells and pathogens at cellular and molecular levels, offering insights into infection dynamics not possible with traditional cell culture models. OoC models allow real-time observation of pathogen invasion, spread through respiratory tissues, cellular responses and immune reactions, highlighting the significance of spatial and mechanical contexts in the progression of respiratory infections.

A foundational study created a biomimetic lung-on-chip model to mimic the alveolar–capillary interface, demonstrating complex responses to bacteria and cytokines. This model showed that simulated breathing motions could amplify the reactions to silica nanoparticles, enhancing cellular uptake and transport [157]. It was also applied to study pulmonary oedema, effectively reproducing drug-induced toxicity mechanisms associated with vascular leakage [164]. Enhancements of the model included integrating TEER measurements for real-time monitoring of epithelial barrier integrity [173]. Additionally, a human small airway-on-chip was developed to study asthmatic responses and COPD characteristics, such as cytokine hypersecretion and increased neutrophil recruitment, especially after viral and bacterial infections [160].

Influenza virus A studies using a human lung airway-on-chip model showed the evolution of resistance to antivirals like amantadine and oseltamivir through sequential passaging, highlighting the model’s utility in studying drug-resistant viral evolution under controlled conditions [174]. Another study with the Influenza H3N2 virus identified mechanical breathing motions as enhancing host defences, significantly reducing viral replication. This effect was linked to mechanosensitive pathways involving TRPV4 and RAGE signalling, offering new therapeutic targets for respiratory infections [170]. Research on viral-induced asthma exacerbation used a human mucociliary airway epithelium model to simulate rhinovirus infection, demonstrating IL-13-induced asthma responses and identifying CXCR2 antagonist MK-7123 as effective in reducing neutrophil migration, suggesting potential for testing immunomodulatory therapies [175]. A human immunocompetent alveolus model explored viral–bacterial co-infections, showing the critical role of tissue-resident macrophages in maintaining barrier integrity and highlighting the significant endothelial damage during co-infections [159]. Upon infection, the model revealed a robust inflammatory response initiated in epithelial cells and extending to the endothelium. Finaly, SARS-CoV-2 infection studies in lung-on-chip revealed rapid endothelial cell impact, with IL-6 elevation indicating a direct viral effect and identifying the cGAS-STING pathway as a key inducer of cell death and a potential therapeutic target [176]. A contributing role of IL-6 in infection-related loss of barrier integrity was confirmed by treatment with tocilizumab, an antagonistic antibody against the human IL-6 receptor. Another study in lung-on-chip models has confirmed the importance of the cGAS-STING pathway in endothelial cells activated by SARS-CoV-2. The pathway induces cell death and type I interferon production. It has been identified as a potential therapeutic target due to its role in triggering abnormal type I interferon responses [177]. These studies collectively underscore the remarkable capability of lung-on-chip to simulate and analyse the complexities of viral respiratory infections, offering unprecedented insights into pathogen dynamics, host responses and therapeutic potentials under physiologically relevant conditions.

### 5.3. Gut-on-chip models

Gut-on-chip models provide a new way to study the complex relationship between the gut microbiota and the human body *in vitro*. These models simulate the oxygen gradients found in the gut microbiota and host tissue, enabling researchers to better understand microbial–host dynamics and their impact on health and disease. Connecting gut-on-chip models and lung-on-chip systems provides insights into how gut dysbiosis relates to respiratory health and identify potential microbial-derived protective metabolites against respiratory viral infections.

Kim *et al*. [178] introduced a gut-on-chip model in 2013, which forms 3D intestinal villi in human Caco-2 cells by exposure to peristalsis-like motions and fluid flow. This model promotes the development of complex villus structures, tight junctions, brush borders, mucus and the differentiation of epithelial cells into types observed in the human intestine [178]. HuMiX is a microfluidics-based system for stable co-culture of human and microbial cells under hypoxic conditions, allowing to study gastrointestinal interactions. In the model, *Lactobacillus rhamnosus* GG and *Bacteroides caccae* induced distinct changes in intestinal cells depending on microbial co-culture [179].

**Figure 6.**
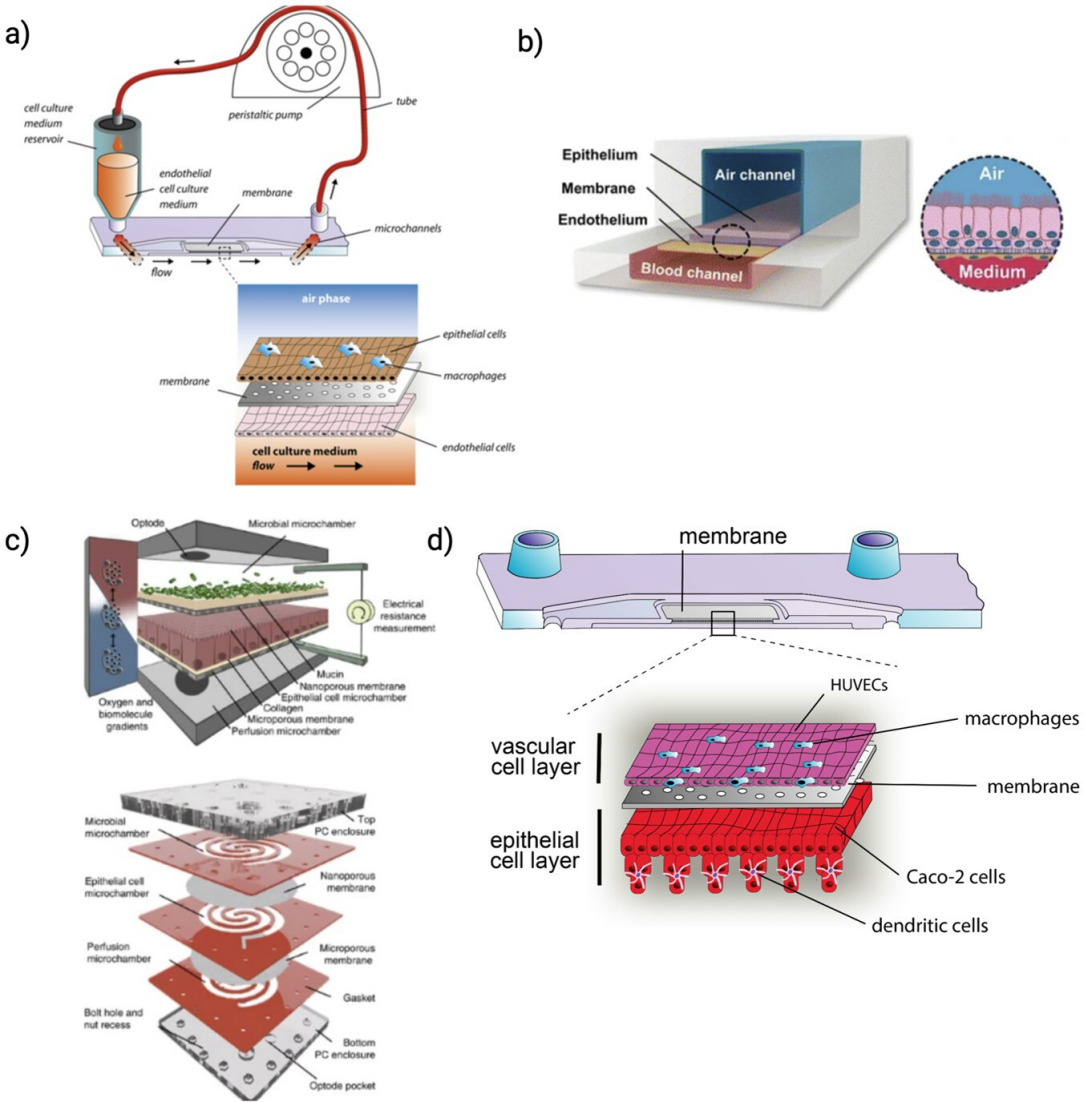
Curated selection of gut-on-chip and lung-on-chip models, highlighting their diverse applications in studying host–pathogen interactions and microbial colonization under physiologically relevant conditions. (*a*) Lung-on-chip model utilized to explore the co-infection mechanisms of influenza virus A and *Staphylococcus aureus* [159]. The model features an air–liquid interface and integrated tissue macrophages to study the host immune response during respiratory infections. (*b*) Small airway-on-chip model designed to mimic the mechanics of breathing. This model is instrumental in studying the physical effects of respiratory movements on lung tissue and pathogen dynamics [160]. (*c*) HuMix model, designed to investigate intestinal microbial colonization in hypoxic conditions, mimicking the gut’s natural environment [179]. (*d*) Immunocompetent model of the human intestine, capable of supporting microbial colonization. This model has been used to study the co-colonization and interactions between the yeast *C. albicans* and the bacterium *L. rhamnosus*, shedding light on microbial interaction and its implications for host tissue integrity and function [181].

The recently developed gut epithelium-microbe-immune (GuMI) microphysiological system has highlighted the progress of studying microbial colonization in gut-on-chip. This system enables the long-term co-culture of oxygen-intolerant commensal *Faecalibacterium prausnitzii*, with human colonic epithelium, antigen-presenting cells and circulating CD4+naive T cells. This system exemplifies the potential of OoC to dissect the contributions of individual immune cells to microbial-induced immune responses, providing a valuable platform for studying gut health and disease [180]. Maurer *et al*. reported a three-dimensional microphysiological model of the human intestine incorporating organotypic structures and tissue-resident immune cells to explore the mucosal immune response to microbial composition changes. This gut-on-chip model supports physiological immune tolerance, allowing for microbial colonization studies, such as the interaction between *Lactobacillus rhamnosus* and *C. albicans*. Pre-colonization with *L. rhamnosus* was found to mitigate *C. albicans*-induced tissue damage and fungal burden [181]. The development of the neonatal-intestine-on-chip represents another significant advancement in modelling intestinal microbial colonization *in vitro* [182]. This *in vitro* model accurately mimics the key features of neonatal intestinal physiology by using intestinal enteroids derived from the intestinal tissue of premature infants, co-cultured with human intestinal microvascular endothelial cells within a microfluidic device. When infant-derived microbiota is introduced, the model successfully recapitulates the primary characteristics of necrotizing enterocolitis (NEC), a fatal gastrointestinal disease affecting premature infants. Mimicked disease hallmarks include the upregulation of proinflammatory cytokines, decreased expression of intestinal epithelial cell markers, reduced epithelial proliferation and compromised epithelial barrier integrity. Finally, Jalili-Firoozinezhad *et al*. [183] presented a gut-on-chip model to maintain complex microbial communities by mimicking oxygen gradients of the human intestinal environment. This approach allowed for the co-culture of aerobic and anaerobic bacteria with human intestinal cells, enhancing intestinal barrier function and preserving microbial diversity. This model replicates physiologically relevant conditions, offering a novel platform for exploring host–microbiome interactions and the potential development of microbiome-based therapies [183]. Further information can be found in other reviews detailing the principles and applications of gut-on-chip models to study microbiome–host interactions [184–187]. A selection of lung-on-chip and gut-on-chip models established for studying host–microbiota interaction is provided in table 2 and figure 6.

**Table 2.** OoC models of lung and gut to study host–microbiota interaction and mechanisms of infection.

study	authors	year of publication	organ model	key findings
reconstituting organ-level lung functions on a chip	Huh *et al*. [157]	2010	lung-on-chip	functional alveolar–capillary interface of the human lung that reproduces organ-level responses to bacteria and inflammatory cytokines
medium throughput breathing human primary cell alveolus-on-chip model	Stucki *et al*. [158]	2018	lung-on-chip	breathing lung-on-chip that mimics alveolar microenvironment and air–liquid interface for long-term co-cultures of alveolar epithelial cells and endothelial cells
co-infection with *Staphylococcus aureus* after primary influenza virus infection leads to damage of the endothelium in a human alveolus-on-chip mode	Deinhardt-Emmer *et al*. [159]	2020	lung-on-chip	alveolus-on-chip to study host–pathogen interactions and identify targets for treatment strategies in pneumonia
invasive aspergillosis-on-chip: a quantitative treatment study of human *Aspergillus* fumigatus infection	Hoang *et al*. [188]	2022	lung-on-chip	aspergillosis-on-chip model for *in vitro* examination of *Aspergillus fumigatus* infection and antifungal drug testing
a new immortalized human alveolar epithelial cell model to study lung injury and toxicity on a Breathing Lung-On-Chip System	Sengupta *et al*. [161]	2022	lung-on-chip	alveolar microenvironment that responds to profibrotic and proinflammatory triggers
gut-on-chip microenvironment induces human intestinal cells to undergo villus differentiation	Kim *et al*. [178]	2013	gut-on-chip	gut-on-chip model, which replicate the differentiated cell types, and physiological functions of normal human intestinal villi, as an *in vitro* model for studying intestinal physiology, digestive diseases and drug development
a microfluidics-based *in vitro* model of the gastrointestinal human–microbe interface	Shah *et al*. [179]	2016	gut-on-chip	gastrointestinal human–microbe model for investigations of host–microbe interactions which can be used in drug screening, drug discovery, drug delivery, pharmacokinetics and nutritional studies
a three-dimensional immunocompetent intestine-on-chip model as *in vitro* platform for functional and microbial interaction studies.	Maurer *et al*. [181]	2019	gut-on-chip	intestinal model for studying microbial communication and host–microbe interactions which can be used to investigate disease mechanisms driven by pathogens
a complex human gut microbiome cultured in an anaerobic intestine-on-chip	Jalili-Firoozinezhad *et al*. [183]	2019	gut-on-chip	intestine-on-chip model that facilitates the extended co-culture of human intestinal epithelium with complex human gut microbiota under controlled oxygen gradients
microfluidic device facilitates *in vitro* modelling of human neonatal necrotizing enterocolitis-on-chip	Lanik *et al*. [182]	2023	gut-on-chip	modelling of necrotizing enterocolitis associated with inflammatory response, dysbiosis, decreased epithelial cell proliferation and gut barrier disruption
an immune-competent human gut microphysiological system enables inflammation-modulation by *Faecalibacterium prausnitzii*	Zhang *et al*. [180]	2024	gut-on-chip	recapitulation of commensal bacterial colonization under hypoxic conditions and its impact on host immune response
modelling of intravenous caspofungin administration using an intestine-on-chip reveals altered *Candida albicans* microcolonies and pathogenicity	Kaden *et al*. [187]	2024	gut-on-chip	intestine-on-chip was used to study fungal interactions and antifungal treatment efficacy on invasive candidiasis
an iPSC-derived small intestine-on-chip with self-organizing epithelial, mesenchymal and neural cells	Moerkens *et al*. [189]	2024	gut-on-chip	IPSC-based intestine-on-chip model comprising myofibroblasts and neurons

### 5.4. hiPSC in OoC: new frontier

Induced pluripotent stem cells (iPSCs) hold great hopes for therapeutic applications, providing advantages over immortalized cell lines or primary cells [184,190]. iPSC can be differentiated into various cell types and provide an unlimited source for generating tissues with a defined composition from a single donor [191]. They are derived from somatic stem cells and express specific markers of crucial transcription factors, including octamer binding transcription factor 3/4 (Oct3/4), determining region Y-box 2 (SRY-Sox2), Krüppel-like factor 4 (Klf4) and cellular-Myelocytomatosis (c-Myc) [192,193]. They can differentiate into specialized cell types derived from the three germ layers ectoderm, endoderm and mesoderm [194]. iPSCs share many characteristics with naturally occurring pluripotent stem cells, including potency, differentiability and the expression of specific stem cell genes, such as embryonic stem cells (ESC) [193]. The discovery of induced pluripotent stem cells (iPSCs) has significantly advanced the field of personalized medicine, allowing for the *in vitro* replication of disease phenotypes that are difficult to model with primary cells due to their limited availability and yield.

Studies indicate multiple pathways causing COPD, with early-life lung development playing a crucial role in determining lung function and the likelihood of developing COPD. Human iPSC (hiPSC) lines derived from COPD patients with severe and early-onset forms of the disease were created, which retain normal karyotypes and the capacity for differentiation into three germ layers [195]. iPSCs thereby offer a personalized approach to study genetic predispositions to diseases and serves as a valuable platform for investigating the mechanisms of infections related to COPD, enhancing our understanding of disease progression and potential therapeutic targets in the context of respiratory viral infections. iPSC-derived lung organoids have been used to model respiratory virus infections and offered insights into the mechanisms of viral infection and pathogenesis, particularly in infant lungs. These organoids mimic the entire developing lung, maintaining the authentic viral genome during infections, unlike viruses grown in standard lab conditions, which often undergo genetic alterations. It was shown that different viruses exhibit distinct interactions with these organoids: the parainfluenza virus sheds without causing morphological changes, the respiratory syncytial virus induces cell detachment and shedding, and the measles virus leads to syncytium formation [196]. Huang *et al*. [197] introduced an *in vitro* human model utilizing iPSC-derived alveolar epithelial type 2 cells (AT2s) adapted to air–liquid interface cultures to simulate early SARS-CoV-2 infection of the lung alveoli. They report rapid transcriptomic changes in infected cells, shifting to an inflammatory phenotype with NF-κB signalling upregulation and a loss of mature alveolar functions. This model validated the efficacy of remdesivir and TMPRSS2 protease inhibition for viral entry, offering insights into cell-intrinsic responses to SARS-CoV-2 and aiding further drug development efforts [197]. A study by Riva *et al*. [198] used iPSC-derived human pneumocyte-like cells to identify potential drugs that could be repurposed to treat COVID-19. Out of approximately 12 000 compounds tested, 100 were found to inhibit the replication of SARS-CoV-2. Among these, 21 showed dose–response relationships. Thirteen of the most promising drugs, including apilimod and various protease inhibitors, were considered likely to achieve therapeutic doses in patients. Critically, the efficacy of these drugs was confirmed in both iPSC-derived human pneumocyte-like cells and a human lung explant model, indicating a fast track to clinical testing due to their existing safety profiles [198]. The development of an hiPSC-derived intestine-on-chip marks another significant advancement in creating a more physiological *in vitro* model for studying developmental biology and personalized therapies. Traditional hiPSC-derived intestinal organoids have limitations due to their closed topology and immature state. However, by employing organ-on-chip technology, researchers have developed a model allowing apical and basolateral access, providing a more accurate simulation of the intestinal environment. In this model, the cells are locally exposed to expansion and differentiation media to replicate growth factor gradients along the crypt–villus axis. This approach enables the intestinal epithelial cells to self-organize into villus-like folds with physiological barrier integrity. Additionally, myofibroblasts and neurons emerge to form subepithelial tissue in the bottom channel, mimicking the natural structure of the human small intestine. The growth factor gradients effectively balance dividing and mature cell types, inducing an intestinal epithelial composition that includes both absorptive and secretory lineages. This composition closely resembles that of the human small intestine [189].

These studies underscore the potential of iPSC-derived models in studying host–pathogen interactions *in vitro*. By differentiating patient-derived iPSCs into relevant cell types, researchers can construct personalized OoC models that accurately reflect individual physiological responses, offering a more precise tool for drug screening and assessing therapeutic interventions. Using autologous models with isogenic patient-derived iPSCs as a source for differentiated lung cell types minimizes the risk of allogeneic reactions that could potentially mask and overlay infection-related mechanisms. It thereby helps to improve the sensitivity and specificity of the model. These models facilitate a deeper understanding of disease progression and response to treatment and support the identification of biomarkers critical for early diagnosis. Moreover, leveraging iPSC-derived OoC models could significantly reduce costs in the drug development process by increasing predictability and minimizing the need for extensive animal testing.

### 5.5. Image analysis for OoC models

Optical microscopy techniques, such as fluorescence microscopy, are essential for studying cellular structures and molecular organizations in OoC models. However, challenges arise in microscopy data analysis. Algorithm-based image analysis combined with systems biology has revolutionized the understanding of OoC models. Traditionally manual and time-consuming, these processes now benefit from advanced algorithms that automatically quantify cellular behaviours and molecular patterns precisely. These machine learning and deep learning frameworks improve accuracy as more data becomes available, facilitating a holistic understanding of biological mechanisms in OoC models [199].

Automated image analysis in OoC models can significantly enhance infectious disease research using sophisticated algorithms to detect, quantify and localize pathogens, thereby improving the accuracy and efficiency in studying host–pathogen interactions [159,181,188,200]. In this approach, standardized pipelines are valuable tools that help to reliably identify infection targets and quantify tissue damage, providing critical data for understanding disease dynamics and testing drug efficacy. Multi-channel 3D imaging and deconvolution processes, though computationally expensive, thereby allow for in-depth analysis of spatial overlaps among cellular components [201,202]. However, efficient data storage and powerful computational resources are necessary to handle the large volumes of generated data.

### 5.6. Standardization of organ-on-chip

Although OoC technology offers promising potential for using lung-on-chip, gut-on-chip and multi-organ-on-chip systems in the context of infection research, standardizing these models for a broader use among the scientific community remains an ongoing challenge.

One of the primary challenges in standardizing OoC models is ensuring reproducibility and robustness across different laboratories. The complexity of these models, which aim to simulate human tissue functionality, structure and interactions, necessitates the development of clear benchmarks for chip fabrication and model assembly. This includes establishing specific guidelines for the materials used in these chips, such as polydimethylsiloxane (PDMS), which, despite its popularity, has limitations like absorption of hydrophobic molecules and protein adsorption, potentially affecting experimental outcomes [203]

It is important to precisely determine airflow and mechanical forces that influence tissue behaviour and pathogen interactions for lung-on-chip models to allow cross-validation of results among labs and platforms. Similarly, gut-on-chip models face unique challenges due to the complex environment of the human intestine, which includes diverse microbiota and varying oxygen levels. Standardization efforts must address the need to replicate these conditions accurately, including the co-culture of human intestinal cells with microbiota under anaerobic conditions. This is essential for studying diseases such as inflammatory bowel disease and enteric infections [204].

**Figure 7.**
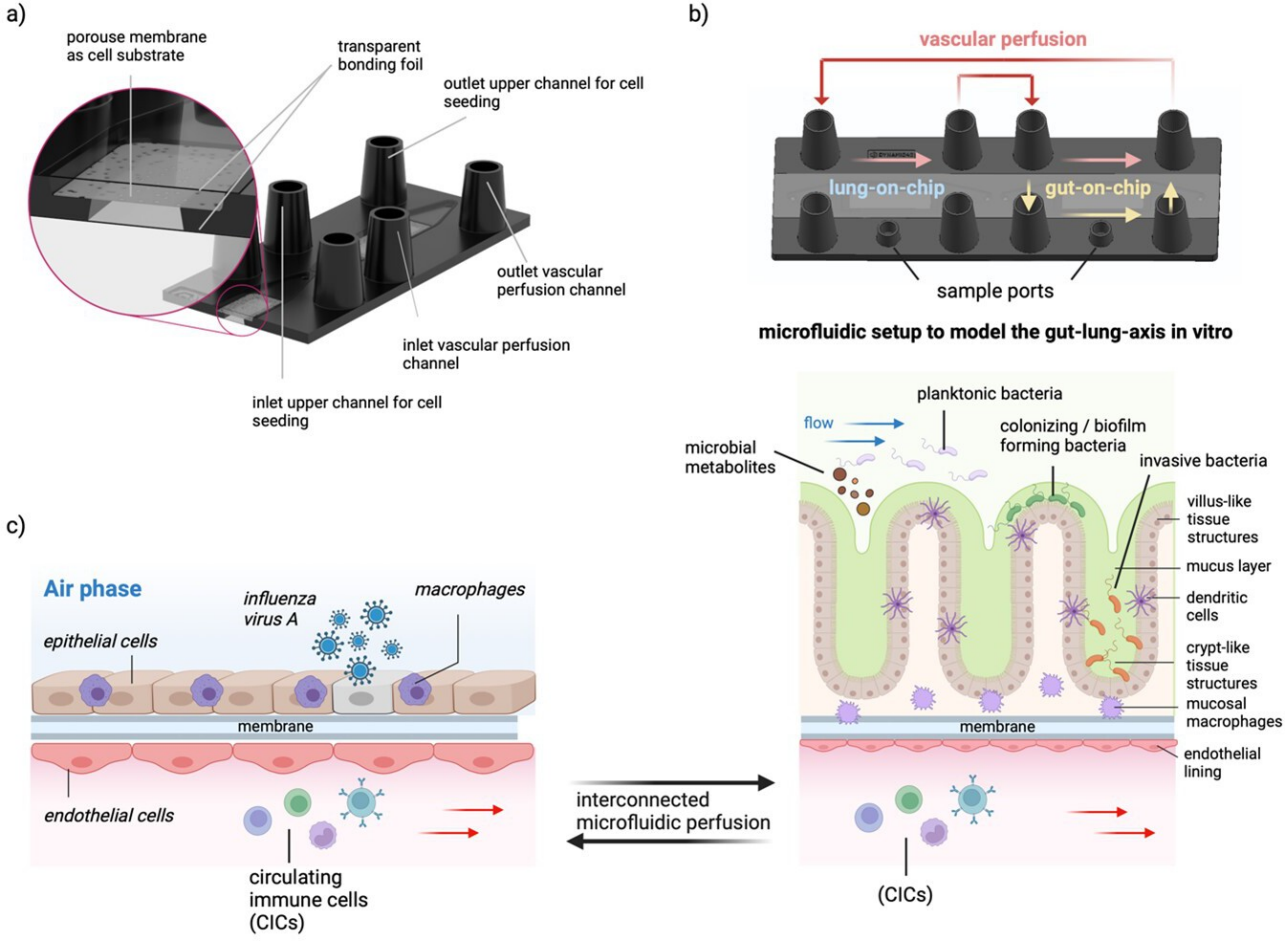
Scheme of a gut–lung axis model utilizing interconnected lung-on-chip and gut-on-chip models via microfluidic perfusion for studying dynamic interactions between the human lung and intestine in a controlled microenvironment *in vitro*. (*a*) Dynamic42 biochip architecture features two separate cavities, each supporting a suspended porous membrane that acts as a substrate for multilayered human lung and intestine organ models. The chip is equipped with Luer-lock ports for independent perfusion of either side of the membranes, facilitating the simulation of distinct organ microenvironments. (*b*) Setup for interconnected organ-on-chip models on the Dynamic42 platform, with the lung model housed in the left cavity and the gut model in the right. A microfluidic perfusion system mimics the physiological blood flow between the lung and gut, establishing a continuous upper flow circuit. This setup ensures the unidirectional perfusion of the gut model’s luminal part to prevent microbial overgrowth while maintaining a non-perfused air phase for the lung model to emulate physiological conditions. (*c*) Showcases the cellular configuration of both organ models, highlighting their interconnection through microfluidic channels that perfuse the endothelial lining and allow the integration and circulation of peripheral immune cells between both organ models. The lung-on-chip model [159] includes epithelial cells and interspersed macrophages exposed to an air phase, whereas the gut-on-chip model [181] contains intestinal epithelial cells, macrophages and dendritic cells with an active luminal perfusion for maintaining homeostatic microbial colonization through removal of overgrowing microorganisms by shear stress. The intestinal model allows to study microorganisms in various states—planktonic, colonizing or invading—alongside microbial metabolites derived from stool sample filtrates, offering insights into the mutual interactions between human tissue and a living microbiota under physiologically relevant conditions (created with Biorender.com).

Multi-organ-on-chip (MOoC) systems offer the advantage of modelling inter-organ communication and systemic responses, which are critical for understanding the pathophysiology of diseases and the pharmacokinetics of drugs (figure 7). However, balancing the conditions for multiple organs within a single chip is challenging. It requires careful optimization of culture media and environmental conditions to support the diverse needs of different tissues. The integration of organ-specific extracellular matrices (ECM) and the use of biocompatible materials can enhance the physiological relevance of these models.

In the context of infection research, OoC models provide valuable insights into host–pathogen interactions, which are often difficult to study *in vivo* due to ethical and technical constraints. However, validation and qualification of these models are essential for their acceptance in both research and regulatory environments. This involves internal validation, which ensures the model’s reproducibility and robustness, and external validation, which confirms that the model’s findings are translatable to real-world scenarios. The establishment of databases such as the Microphysiological Systems Database, which compiles *in vitro* and *in vivo* data, supports the external validation of OoC models by allowing comparisons with clinical data [205].

The development of standardized protocols and guidelines, such as those proposed by the European Committee for Standardization (CEN) and the European Committee for Electrotechnical Standardization (CENELEC), along with initiatives like the ORCHID project, are crucial steps towards achieving these goals [206]. These efforts aim to harmonize the design, fabrication, operation and reporting of OoC experiments, thereby facilitating the reproducibility and scalability of these models.

Another challenge remains in sample variability from OoCs, arising from differences in immune cell donors, cell types or testing conditions, which complicates image analysis. Machine learning and deep learning algorithms can address these challenges by identifying complex relationships between image features and biological characteristics [176,207]. However, obtaining large annotated datasets remains labour-intensive.

For acquiring high-quality image data, advanced microscopy approaches like super-resolution and multimodal microscopy offer a high spatial and temporal resolution, essential for studying, i.e. viral infections in OoCs. Techniques like STED, STORM, PALM and SIM allow molecular-scale imaging below conventional resolution limits [208]. Accurate labelling and recent advancements in live-cell tagging and immunolabelling enhance the imaging of deep cell layers [209–211]. Combining such optical recordings with methods like Raman spectroscopy further enhances options for even label-free tissue characterization [212].

Image-based systems biology integrates automated image analysis with spatio-temporal modelling for predictive simulations. This approach quantifies biological processes and helps to understand dynamic cellular interactions and mode of action of therapeutic interventions. In this context, deep learning algorithms have improved the quality of microscopy imaging in OoCs, enhancing spatial resolution and reducing data volume [213,214].

Integrating image analysis in lung-on-chip and gut-on-chip studies has provided insights into mechanisms underlying diseases like COPD and infections with *Staphylococcus aureus* or influenza A [159,160,215]. Advanced image analysis has further quantified aspects of *Aspergillus fumigatu*s infection in lung-on-chip models, contributing to understanding the disease and testing antifungal treatments [188]. Quantitative data from image-based systems biology enable virtual infection scenarios, advancing our understanding of host–pathogen interactions in respiratory viral infections.

In summary, OoC models, combined with advanced microscopy and image analysis, offer transformative approaches to studying gut–lung interactions in respiratory viral infections. These technologies provide comprehensive insights into disease mechanisms and treatment effectiveness, advancing our understanding of microbiome influences on respiratory health.

## 6. Conclusion and future prospects

The lung microbiome has emerged as a key player in modulating immune responses and disease susceptibility, highlighting the importance of microbial balance for respiratory health. As research continues to evolve, novel methods for a detailed lung microbiome analysis promise to further enhance our understanding of respiratory infections. OoC models have proven invaluable for simulating the complex architecture and mechanical forces of the lung, enabling detailed studies of viral infections, drug responses, and the gut–lung axis in a controlled environment. Meanwhile, hiPSCs offer unprecedented opportunities for personalized medicine, enabling the development of patient-specific models to study disease mechanisms and test therapeutic interventions. This multidisciplinary approach will undoubtedly pave the way for novel therapeutic strategies and preventive measures, ultimately improving outcomes for patients with respiratory diseases.

**Figure 8.**
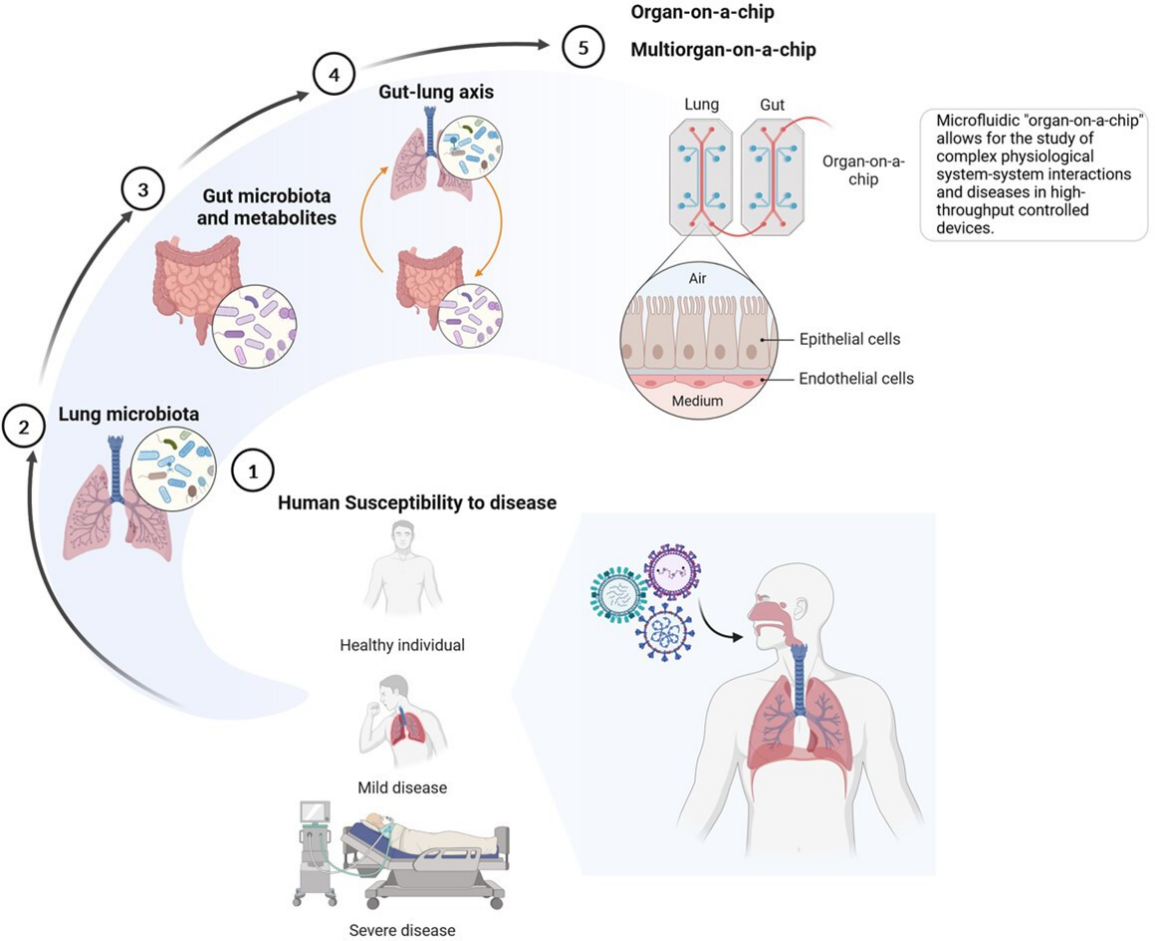
There are many diverse causes underpinning human susceptibility to disease (1). These include respiratory disease provoked by influenza A virus, RSV and SARS-CoV-2 as discussed in this review. Among these is a multi-layered complex parameter that relates to (2) the lung microbiome and (3) the gut microbiome. (4) The gut–lung axis regulates human homeostasis and susceptibility to viral infection evolution. Some molecular mechanisms have been identified and explored in this review, such as how microbiota and its metabolites prime the immune system, regulate the growth of selected microbial populations, and how lung or gut dysbiosis affects this equilibrium. However, many other processes are challenging to investigate, as we are still unable to fully capture the microbial populations and their metabolites in these tissues, and we lack details on how different microbial populations interact with each other. Understanding such interactions is critical for controlling human disease. (5) Organ-on-chip technology offers a controlled way to investigate these complex, multi-layered interactions in human-centred physiological contexts that take into consideration the tissue, innate barriers (including mucous), different cell types resulting in an epithelial layer separated from endothelial cells that create the air–liquid interface and can also include the immune system (created with Biorender.com).

Exploring the lung microbiome and the dynamics of viral infections with OoC technology offers a unique opportunity to dissect further the mechanisms of the gut–lung axis in health and disease and to study the impact of gut microbiota on lung immunity and inflammation. This insight is critical for elucidating the complex interplay between different organ systems and their contribution to respiratory health. These systems will be valuable tools to unveil the complex dynamics of the lung microenvironment and its impact on respiratory health, offering new insights into host–pathogen interactions and the mechanisms underlying respiratory diseases (figure 8).

Moreover, OoC models serve as an innovative drug-testing platform with significant potential for evaluating the efficacy of antiviral drugs and developing preventive strategies against respiratory viral infections. These systems can provide a more accurate and predictive model platform for assessing personalized drug responses, enabling the identification of correlates of protection essential for vaccine development and therapeutic interventions. The precision and control afforded by these models facilitate the rapid screening of potential treatments, accelerating the process from research to clinical application.

To improve our ability to predict respiratory disease progression, identify novel therapeutic targets and develop more effective treatments for respiratory diseases, it is important that future research focuses on further integrating various technologies. By addressing the remaining technical challenges and leveraging the opportunities for innovation, OoC models can provide more reliable and predictive insights into human diseases. By doing so, more comprehensive *in vitro* models of respiratory diseases (i.e. studying inter-organ communications and systemic responses to respiratory infections) can be created. These models can help us gain a better understanding of complex biological phenomena and can contribute to significant breakthroughs in respiratory disease research and treatment. This will ultimately support the development of new therapies and reduce the reliance on animal testing.

## Data Availability

This article has no additional data.
